# Cardiovascular Disease Burden among African Migrants

**DOI:** 10.1007/s11883-025-01307-w

**Published:** 2025-05-30

**Authors:** Karlijn A.C. Meeks, Charles Agyemang

**Affiliations:** 1 Division of Endocrinology, Diabetes and Nutrition, Department of Medicine, University of Maryland School of Medicine, 670 W. Baltimore Street, HSF III 4061, Baltimore, MD 21201 USA; 2 Department of Epidemiology and Public Health, University of Maryland School of Medicine, Baltimore, MD USA; 3 Department of Public and Occupational Health, Amsterdam UMC, University of Amsterdam, Amsterdam Public Health Research Institute, Meibergdreef 9, Amsterdam, 1105AZ the Netherlands; 4 Division of Endocrinology, Diabetes and Metabolism, Department of Medicine, Johns Hopkins University School of Medicine, Baltimore, MD USA

**Keywords:** Cardiovascular Disease, African Migrants, Migration, High-Income Countries, Cardiometabolic Risk

## Abstract

**Purpose of Review:**

To provide an overview of the current available evidence on the burden of cardiovascular diseases (CVD) among African migrants, including its risk factors, underlying mechanisms, and prevention and treatment efforts, while highlighting critical gaps in knowledge.

**Recent Findings:**

The CVD burden is high among most African migrant populations. Underlying mechanisms for the high CVD burden include various pre- and post-migration factors, genetics, and epigenetics. Studies increasingly show substantial variation in CVD burden among African migrants across factors such as country of origin, host country, reason for migration, duration of stay, sex, and age. This variation is also observed among CVD risk factors and requires tailored prevention and treatment efforts.

**Summary:**

To fill critical gaps in knowledge, future studies need to recruit among diverse African migrant populations, in various high-income countries, using standardized methodologies with a focus on longitudinal designs, and integrating lifestyle, sociocultural, environmental, and genetic factors.

## Introduction

Cardiovascular diseases (CVD) pose a major threat to human health, but the burden is not evenly distributed across world regions and population groups [1]. While the majority of new CVD cases globally occur in individuals aged 50 and older, the age profile is markedly different in sub-Saharan Africa, where 40% of cases affect individuals under 50 years of age, with ischemic heart disease being the most common condition in this age group, followed by stroke and hypertensive heart disease [2]. Alarmingly, in Eastern and Central Africa, 30% of incident cases occurred in people younger than 25 years.

Migrants are one such population group often differentially affected by CVD [3]. Migration has the potential to profoundly shaped human health, including cardiovascular health. At its core, migration is the movement by people from one place to another with the intension of settling temporarily or permanently in the new location [4]. Most international migrants relocate for work, family reunification, or education and are often referred to as economic migrants. In contrast, individuals forced to leave their homes due to persecution, conflict, or natural disasters are considered refugees. When refugees seek formal protection in another country, they become asylum seekers.

**Fig. 1:**
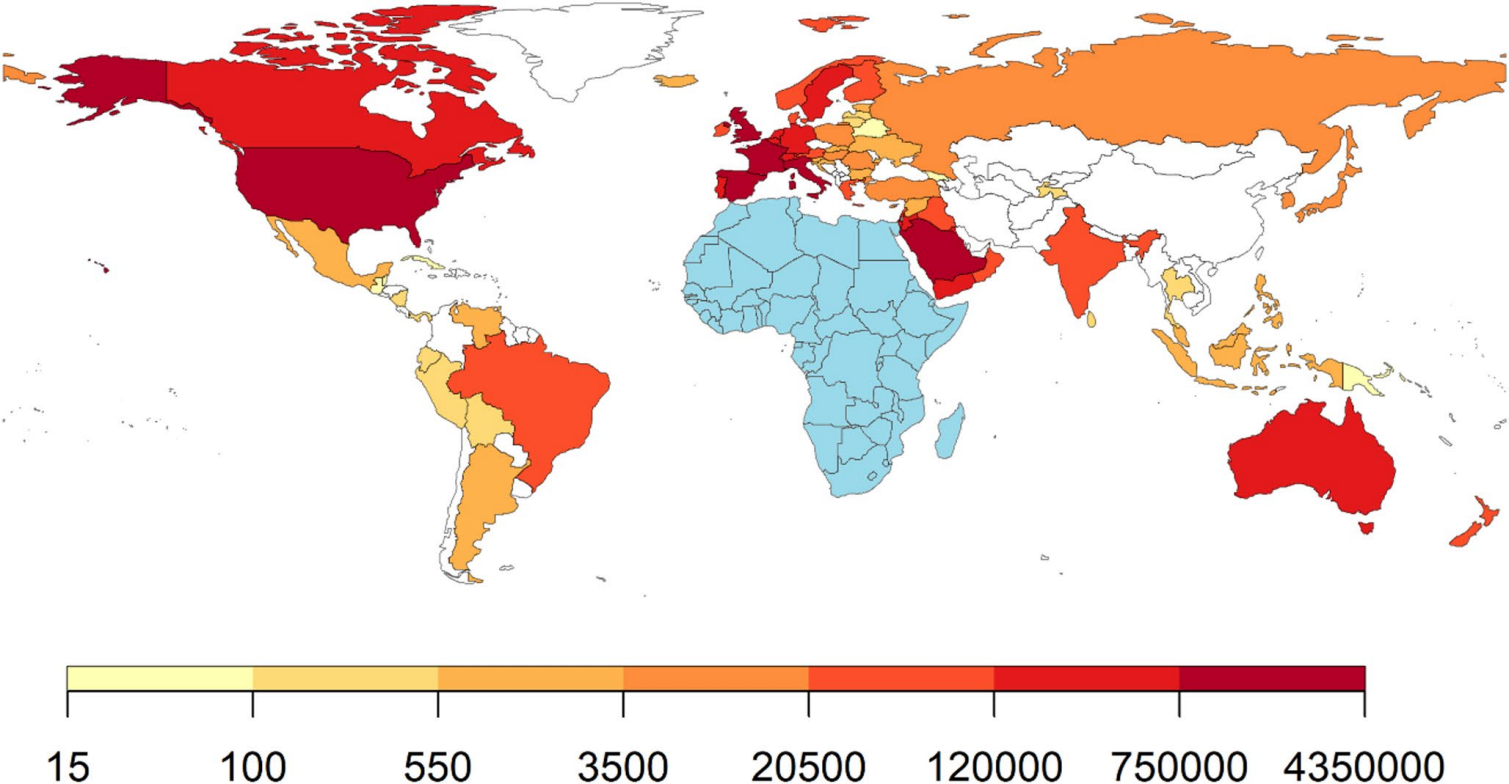
Distribution of African migrants to countries outside the African continent. Data source: UN DESA 2020 [5]. No data were available for countries in white

There were an estimated 281 million international migrants in the world in 2020, accounting for about 3.6% of the world population [4]. An approximate 14% of all international migrants in 2020 originated from Africa, representing 40.5 million people [5]. Notably, about 20.9 million of these people migrated to other African countries. Outside of Africa, North America and Western Europe are the most common destination regions for African migrants with France, the United States (US), and the United Kingdom (UK) being the top three destination countries (Fig. 1).

Although African migrants represent a large share of the world migrant population, up-to-date knowledge on the CVD burden among this population is limited. Addressing CVD in African migrants requires targeted action, which in turn depends on a thorough understanding of the prevalence, risk factors, and underlying mechanisms driving CVD in this population. This review provides an overview of the current available evidence and highlights critical gaps regarding the burden of CVD and its determinants among African migrants. We focus specifically on African migrants who have migrated out of the African continent as data on this population is particularly limited.

## CVD Burden in African Migrants

The cardiovascular health status of African migrants living outside of Africa is understudied, with available studies varying widely in design, methodologies, and findings. Despite this heterogeneity, emerging evidence underscores a substantial CVD burden in this population.

African migrants in Europe have been shown to experience disparate CVD outcomes depending on the country of residence, region of origin, and the host population used for comparison. For instance, higher rates of coronary heart disease (CHD) and stroke were observed among African migrants in Portugal compared with the host population [6]. In Italy, the risk for hospitalization for stroke was highest in sub-Saharan African migrant men and women compared with both Italian born natives and migrants from all other world regions [7]. Conversely, a lower risk of CVD events was reported among African-ancestry compared with European-ancestry individuals in the UK Biobank, yet most of this difference was explained by environmental factors, such as employment status, social deprivation, qualifications, and household income [8]. Moroccan migrants in the Netherlands were found to have lower incidence rates of stroke compared with the Dutch host population [9]. In terms of mortality, some of the highest CHD mortality rates have been reported for East African migrants in England and the lowest for North Africans in the Netherlands [10, 11].

Research on African migrants in the US also reveals significant variability in CVD risk and outcomes [12]. For instance, while most studies on African refugees showed an increased risk of CVD and its risk factors [12], other studies show more favourable outcomes. Among Nigerian and Ghanaian migrants in the greater Washington DC area, the distribution of CVD risk resembled that of the general US population [13]. When compared to US-born African-ancestry individuals, African migrants were found to have lower cardiovascular mortality and comparable stroke mortality rates [14]. Similarly, a study comparing African migrants to African Americans and European Americans found African migrants to have lower odds of stroke than African Americans and similar odds to European Americans [15]. This highlights the heterogeneity in risk profile across African-ancestry populations living in high income countries and that findings from African Americans, for example, should not be extrapolated to African migrants.

While comparison with host population of the country of destination is the most common study design for migrant health studies, valuable additional insights regarding the role of migration can be obtained when comparing migrant populations with compatriots still living in the country of origin [16]. The Research on Diabetes and Obesity among African Migrants (RODAM) study of Ghanaian migrants in Europe revealed a significantly increased risk of CVD compared with Ghanaians living in Ghana [17].

## Risk Factors for CVD in African Migrants

### Cardiometabolic Risk Factors

Meta-analysis of 35 articles reporting on African migrants in high-income countries showed that the most prevalent cardiometabolic risk factors were obesity (59%), dyslipidaemia (29%), hypertension (27%), and type 2 diabetes (T2D) (11%) [18]. Of note, 77% of the articles in this meta-analysis reported on East African migrants with Somalian migrants the most studied group, while the burden of these risk factors varies significantly based on factors such as country of origin, destination, sex, reason for migration, and duration of stay.

#### Obesity

The prevalence of obesity among African migrants is generally high. A national survey on African migrants in the US reported a combined overweight and obesity prevalence of 65% [19]. Among West African women living in Western Australia, 80% were found to be overweight or obese compared with 49% of Australian women [20]. A high prevalence was also reported for Ghanaian women in Eastern Australia (66% overweight or obese, 26% obese) [21]. Data from the RODAM study demonstrate regional variation among a homogenous group of African migrants [22]. The prevalence of obesity among Ghanaian migrant women in London was 54%, compared with 49.4% among Ghanaian women in Amsterdam, and 39% among Ghanaian women in Berlin. A similar pattern was seen in men, but at lower rates; 21% for Ghanaian men in London, 19% in Amsterdam, and 14% in Berlin [22].

Studies show an alarming increase in obesity among Somalian refugees over time. In 2001, a small study in New Zealand reported obesity for 19% of Somali refugee women [23]. Among Somali migrants in Oslo, a 2010 study reported that 15% were obese and 35% were overweight [24]. Five years later, a similar study among Somali migrants conducted between 2015 and 2016, found 9.2% of men and 44.1% of women obese [25]. Similarly, a study recruiting Somali migrants in the same years in the US found a much higher prevalence of obesity of 24% in men and 57% in women [26].

North African migrants in Europe also exhibit a wide range of obesity rates, from 3.6% to 39%, as seen in a systematic review of studies conducted between 1999 and 2010 [27]. More recent data from Amsterdam (2011–2015) showed an obesity prevalence of 19% in Moroccan men and 35% in women, reflecting a global rise in obesity over time [28]. Duration of residence often exacerbates this trend. For example, 20% of North African migrant women in Italy who had lived there for less than 10 years were obese, compared to 37% for those residing over a decade [29].

#### Dyslipidaemia

Notable variations in major clinically measured lipids - high-density lipoprotein (HDL), low-density lipoprotein (LDL), total cholesterol, and triglycerides - have been observed in African-ancestry populations compared with other populations [30]. West Africans both in Africa and in the diaspora generally have a more favourable lipid profile even when facing a higher burden of CVD and cardiometabolic risk factors than other population groups [30]. In contrast, disproportionately high triglycerides have been reported among East Africans [31]. While an unfavourable lipid profile is considered a risk factor for CVD across populations, there is evidence that the strength of association between lipids and cardiometabolic risk factors may differ across African populations [31]. However, the effect of lipids on stroke risk was found to be similar between African ancestry individuals and European ancestry [32].

Out of the major lipids, the prevalence of elevated LDL cholesterol was highest in Ghanaian migrant men and women in London (59.9%, 53.6%), followed by elevated total cholesterol (47.6% and 47.8%), low HDL (15.1% and 22.8%), and elevated triglycerides (7.4% and 4.2%) [33]. A similar pattern was observed among Ghanaian migrants in Amsterdam and Berlin. Urbanization that accompanies migration seems to play a major role in the dyslipidaemia burden among African migrants. Dyslipidaemia prevalences were substantially less favourable among Ghanaian migrants in Europe compared with peers living in rural Ghana [33]. Ethiopian African migrants in Minnesota had a lower prevalence of elevated LDL and total cholesterol (10–20%) compared with these Ghanaian migrants in Europe, but elevated triglycerides was more common (18–22%) as was low HDL (20–37%) [34]. This is in concordance with the high triglyceride levels observed in the East Africa region. Somali migrants in the US, many of whom refugees, had an overall more unfavourable lipid profile with a high prevalence of low HDL (32–49%), and elevated total cholesterol (49–63%) [26].

Lipoprotein(a) [Lp(a)] is a type of lipid that has emerged as an independent risk factor for atherosclerotic CVD with higher circulating levels in African-ancestry individuals. Analysis of UK Biobank data revealed that while Africans living in the UK had higher absolute Lp(a) levels compared with other ethnic groups, the relative atherosclerotic CVD risk gradient across Lp(a) levels was similar [35]. However, studies on Lp(a) among diverse African migrant populations are limited and more work is needed to determine the potential role of Lp(a) in CVD risk among African migrants residing in diverse environmental contexts.

#### Hypertension

In meta-analysis, the pooled prevalence of hypertension among sub-Saharan African migrants in Europe was found to be higher than in European host populations and other migrant groups, such as South Asians [36]. Notably, studies have reported higher rates for West African migrants than East and North Africans. For example, among Ghanaian migrants in Europe, hypertension prevalence was reported at 59.5% for men and 58.5% for women [37]. The prevalence of hypertension among Senegalese migrant men in Italy was 35%, higher than that observed among migrant men from Morocco(18.2%), Tunisia (22.2%), and Pakistan (15.4%) [38]. Somali migrant men and women had a lower prevalence of hypertension (19.0% men, 13.6% women) compared with the Finnish host population (36.0% men, 23.9% women) [39].

In North America, the prevalence of hypertension among African migrants appears lower than in Europe, in concordance with a lower prevalence of hypertension among the general population of North America compared with Europe [40]. Data from the National Health Interview Survey (NHIS) in the US show a hypertension prevalence of 23% among African-born individuals, lower than the 37.8% observed among African Americans [19]. Notable variability exists also in this region depending on country of origin and sex. Among Ethiopian migrants in Minnesota, 24% of women and 33% of men had hypertension [34]. Similarly, for Somalians, another major East African migrant group in the US, a 21% hypertension prevalence was observed among women and 29% among men [26]. Rates were also comparable for migrants in the US originating from West and Central Africa (32%) [41], and Southern Africa (34.7%) [42].

Outside North America and Europe, data are sparse and highly variable. In Melbourne, Australia, hypertension prevalence among African migrants was 16% [43]. In Sydney, reported hypertension rates for Ghanaian migrants were higher for men (40%), than for women (17%) [21]. Among Ethiopian migrants in Israel, the prevalence was 18.6%, comparable to the Israeli host population at 17.5% [44].

#### Type 2 Diabetes

The burden of T2D is also markedly high among African migrants, with prevalence rates generally exceeding the global estimate of about 9% of the world population living with T2D [45].

Pooled, sub-Saharan African migrants had 2.6 times higher odds for T2D compared with the European host population [46]. Notable differences are observed by country of birth and country of residence. For instance, Ghanaian migrants had prevalence rates of 15.3% in men living in Berlin, 12.8% in Amsterdam, and 10.4% in London, with similarly high rates among women: 10.2%, 9.9%, and 8.4%, respectively [22]. In Sweden, 4.4% of sub-Saharan African migrants had T2D and 16.8% of North African migrants, compared with 2.5% of Swedish born people [47]. The highest T2D mortality ratios have been reported for North African women in Spain [48].

Data on African migrants from the US show a similar picture. In a nationally representative sample, 7.9% of participants born in Africa had T2D [19]. Differences between economic migrants by region of origin and destination seem minimal as 12.6% of West African migrants in Baltimore had T2D compared with 9–12% of Ethiopian migrant men and women in Minnesota [34, 41]. Somali refugees in the US on the other hand exhibited even higher rates, with 14.6% of women and 21.1% of men affected by T2D [26].

### Behavioural Risk Factors

The WHO recognizes unhealthy diet, physical inactivity, tobacco use, and harmful use of alcohol as the most important behavioural risk factors of CVD. These behavioural risk factor influence CVD risk either directly or mediated via the cardiometabolic risk factors discussed above.

#### Physical Activity

African migrants in many settings face challenges in meeting recommended physical activity levels. In the US, 57% of African migrants did not meet the physical activity criteria, which is high, but very similar to African Americans (56.7%) [19]. In Europe, physical inactivity rates different by host country. Among Ghanaian migrants in London 36.6% did not meet physical activity recommendation, compared with 24.1% in Berlin and 14.6% in Amsterdam [49]. In Sweden, physical activity patterns among Somali migrants appeared to vary by BMI. Among those with a lower BMI, 34.5% met the WHO recommendations for physical activity, compared to just 17.5% of those with a higher BMI [50]. In France, hypertensive patients from North Africa were less likely to be physically active compared with native French patients [51].

#### Diet

Diet is often subject to substantial change upon migration from Africa to other world regions [52]. Some African migrant populations seem to adhere to healthier dietary practices from their home country, while others adapt unhealthy dietary habits of the host country [53].

Tunisian migrants in France scored higher than the French host population on a diet quality index and relatedly had a lower prevalence of cardiometabolic risk factors, suggesting that preserving healthy dietary characteristics after migration contributes to a favourable health effect in some African migrant groups [54]. In contrast, eating habits of African migrant students in Nanjing, China, were found to be less healthy compared to their counterparts in their countries of origin (Lesotho, Nigeria, Ghana) [55]. While frequent consumption of snacks and high energy dense foods was reported among Somali migrants in Sweden, there were no differences reported in dietary habits in relation to BMI in this African migrant population [50]. In Spain, about 70% of migrants from Equatorial Guinea adhered to a dietary pattern labelled as “Western”, which was characterized by energy-dense, fat-rich foods [56]. A healthier, more traditional dietary pattern was consumed by migrants who were on average older and had a longer duration of stay. This suggests that younger people and more recent migrants are more readily adopting a Western dietary pattern than older peers and peers with a longer duration of stay.

#### Smoking

Smoking contributes to CVD among African migrants, but the degree differs per migrant group [57]. Among Moroccan and Ghanaian migrants in the Netherlands, smoking-related CVD risk is lower in women (0.1% and 4.3%) compared with men (10.8% and 8.8%) [57], mirroring the substantially lower smoking prevalence among African migrant women compared with men [58]. This suggests that smoking is a greater CVD risk factor among African migrant men than women. In North America, smoking may be less of a contributor in African migrants compared with other US populations as smokers made up 4.3% of African migrants in the US compared with 18.6% of African Americans [19]. A study in Minnesota reported a prevalence of 8.3% ever smokers and found no significant differences based on country of origin, which included Somalia, Ethiopia, Libera, Sudan, and Kenya [26].

#### Alcohol

Excessive alcohol consumption has long been recognized as a behavioural risk factor for CVD. While there has been controversy on the role of light to moderate use of alcohol, recent studies indicate increased CVD risk at all levels of alcohol consumption [59]. In the US, studies report low rates of alcohol consumption among sub-Saharan African migrants [53]. However, alcohol consumption is very variable depending on the circumstances of migration. Rates of excessive alcohol use are higher among forced migrants for whom alcohol use can be a form of coping with traumatic experiences [60]. Other factors contributing to variation in alcohol use include religious affiliation and time since migration [61].

## Underlying Mechanisms of the CVD Burden in African Migrants

The potential factors underlying the disparate burden of CVD in African migrants are multifaceted including premigration and postmigration factors, genetics, and epigenetic modifications among many other factors [62].

### Pre-migration Factors

Evidence indicates that early life factors such as inadequate maternal nutrition, low birth weight, intrauterine growth restriction, and childhood malnutrition influence the development of CVD later in life [63]. Low birth weight, for example, has been shown to be associated with changes in cardiovascular structure and function, including changes in regulation of blood pressure, impaired vascular endothelial function, and unfavourable lipid profile [63]. Poor early life factors can also affect metabolic pathways, resulting to insulin resistance, disrupted glucose metabolism, and increased adiposity, and subsequent increase in the risk of CVDs [63]. The RODAM study among Ghanaian migrants in Europe found early life factors to be associated with estimated 10-year atherosclerotic cardiovascular disease [64], metabolic syndrome [65], and T2D [66]. Economic challenges in several African countries have exposed many migrants to adverse early life circumstances such as limited access to prenatal and childhood health care, inadequate nutrition and poor socioeconomic conditions, increasing risk of CVD in adulthood upon migration [67]. This phenomenon is line with the thrifty phenotype hypothesis, which suggests that people who are exposed to poor early life circumstances develop an adaptive “thrifty” phenotype that becomes disadvantageous in obesogenic environments, commonly seen in many high-income countries [68]. This clearly highlights the importance of taking early life factors among migrants into consideration when assessing migrants’ health.

In contrast, when African migrants exhibit a more favourable CVD risk profile compared with host populations, such as lower stroke prevalence and mortality among African migrants in the US, it has traditionally been attributed to the “healthy migrant effect”. This hypothesis ascribes reduced risk of certain CVDs and other non-communicable diseases to a selection effect with healthier individuals more likely to migrate [69]. However, this widely cited hypothesis has been challenged as it is difficult to test and there is a need for more studies linking both health advantages and disadvantages to specific environmental exposures, such as lifestyle factors [70].

### Post- Migration Factors

Postmigration factors such as socioeconomic status (SES), work and occupation, neighbourhood environment, social support, cultural factors, and access to health care, can all influence the health-related behaviours and cardiovascular outcomes among migrants. Migration to high-income countries often creates economic challenges for migrants as many carry the economic burden of looking after themselves in the destination countries as well as their families left behind in their countries of origin [71]. This challenge may introduce financial stress, which may create barriers in accessing preventive services, healthy foods, heightened psychosocial stress levels, and subsequently increase the risk of CVD [72, 73]. In addition, many African migrants engage in physically demanding jobs often accompanied by exposure to occupational hazards, which can directly or indirectly affect their CVD risk [74, 75]. Furthermore, migrants often congregate in disadvantaged neighbourhoods in the destination countries with limited access to fresh, nutritious foods; safe recreational areas; and health care facilities [76], which may contribute to poor dietary choices, physical inactivity, and psychosocial stressors, all of which increase their risk of CVD [77]. In addition, cultural beliefs and practices can significantly influence health care–seeking behaviours, adherence to medical advice, and the use of preventive care services and subsequent cardiovascular health outcomes [78]. Finally, barriers to health care access, often due to lack of health insurance, transportation difficulties, unfamiliarity with the health care system, or distrust in health care system, can result in delays in seeking medical care [79, 80], including preventive care and regular screenings for cardiovascular risk factors. This can lead to undiagnosed and untreated conditions that contribute to CVD [81].

Post-migration factors that may contribute to the favourable CVD profile observed in some African migrants include maintenance of a healthy traditional diet with low alcohol intake and gaining access to preventive services that were less available in their country of origin [82]. However, evidence suggests that this CVD advantage, if present, tends to diminish over time. For instance, a Dutch study that compared incidence rates of first acute myocardial infarction among ethnic groups in the Netherlands found that Moroccans had a significantly lower incidence compared with the Dutch host population in the initial phase, but this lower incidence disappeared within ten years since migration [83].

### Genetics/Epigenetics

Genetics can influence cardiovascular health in profound ways and can affect most cardiometabolic risk factors [84]. Studying the genetics of CVD risk is particularly of interest for African populations because of the high level of genetic diversity among populations within Africa [85]. While most disparities between African migrants and host populations are driven by environmental and social factors, a comprehensive understanding of CVD in African migrants requires studying genetics and its interaction with environmental influences.

*APOL1* risk variants are a striking example of genetics influencing differential CVD risk in African-ancestry populations. These variants, common in some African populations but are virtually absent in populations without genetic African ancestry, were positively selected for protection against sleeping sickness [86]. In a recent analysis among people with recent African ancestry living in the UK, *APOL1* genotypes - initially identified in relation to chronic kidney disease - were found to be associated with 27 additional conditions, including CVD [87].

Another clear example is the A- haplotype of the *G6PD* gene, which is estimated to be present in 10% of sub-Saharan Africans [86]. It rose to that high frequency in certain parts of Africa because it offers protection against malaria. Recent work has shown that African migrants carrying this haplotype had almost a full percentage point lower mean HbA1c than those who didn’t, due to haemolysis and faster red blood cell turnover caused by G6PD deficiency [88]. The resulting lower HbA1c levels are independent of the degree of glycemia, contributing to underdiagnosis of T2D in African migrants.

Epigenetics, which studies DNA modifications without sequence changes, is also gaining recognition in migrant health research [89]. The majority of epigenetics studies have been performed in European-ancestry populations, with studies among sub-Saharan Africans especially scarce [90]. Epigenome-wide association analyses comparing Ghanaian migrants with non-migrants identified 13 epigenetic markers differentially methylated, many of which could be linked to CVD and related diseases [91]. Furthermore, some epigenetic markers have been associated with perceived discrimination among African migrants, suggesting that epigenetics may serve as a mechanism through which migration-related stressors influence CVD risk [92].

African-ancestry populations remain significantly underrepresented in genomics and epigenomics research. To date, there is no GWAS for CHD in sub-Saharan Africans, even though a large number of SNPs identified in ancestry-specific cohorts indicates the potential value of conducting such studies in this population [93]. Greater inclusion is essential to ensure that African migrants benefit equitably from advances in genomic medicine.

## Prevention and Treatment of CVD in African Migrants

### Prevention

The primary prevention of CVD begins by identifying the key risk factors, especially modifiable risk factors, to help target therapeutic lifestyle changes [94]. This is highly relevant because modifiable risk factors account for about 50–70% of CVD events and deaths [95, 96]. Lifestyle intervention involving minimizing weight gain, adopting a healthy diet, ensuring adequate sleep duration, engaging in regular physical activity and avoiding smoking can prevent CVD and related complications [97]. Because of the high prevalence of obesity and hypertension in African migrant groups, weight reduction programs that provide African migrant groups specific dietary advice and promote physical activity as well as low salt and high potassium diet may be especially helpful in reducing obesity and hypertension related complications. Community engagement interventions have a positive effect on lifestyle interventions and health cardiovascular outcomes. However, intervention trials targeting CVD prevention failed to include diverse populations including African migrant populations and it thus remains unclear whether the results from these trials can be extrapolated to African migrants with different cultural traditions and lifestyles. As a result, evidence on how best to deliver effective culturally adapted health promotion interventions to prevent CVD in African migrants is lacking. Moreover, evidence on effective implementation strategies, effectiveness and cost-effectiveness of these health promotion interventions among African migrants are currently also lacking and clearly highlights the urgent need to validate these interventions in migrant communities, taking into account the context in which they live.

### Treatment

Patients with established CVD risk factors have a high risk of subsequent CVD events, including myocardial infarction and stroke. For these patients, therapeutic lifestyle changes of proven benefit, including dietary modification/weight loss, increased physical activity, and smoking cessation, can improve outcomes. Additional measures include treatment of the risk factors such as hypertension, dyslipidaemia and T2D. However, evidence shows suboptimal control of CVD risk factors in African migrant groups in spite of their high levels of awareness and treatment rates [98, 99]. This has been suggested to be due to poor adherence to treatment and lifestyle recommendations, possibly due to low health literacy, poor care standards, poor quality of care or ineffective response to glucose-lowering agents [99, 100]. This finding clearly suggests the need for greater efforts to improve the effectiveness of CVD risk factor management in African migrants. Large gains can be achieved in African migrants by removing barriers that prohibit effective CVD risk management, particularly physician inertia and nonadherence to treatment recommendations. More emphasis should be on increasing awareness, identification of barriers for improving lifestyle, and patient education. Patients should be provided information through trusted sources such as through community leaders regarding CVD-associated risks, benefits of treatment, and possible side effects in a way that can be understood taking into account language barriers, as well as cultural and socioeconomic background. In a culturally adapted hypertension education (CAHE) to improve blood pressure control and treatment adherence in patients of African origin, including African migrants led to significant improvements in blood pressure control and adherence to lifestyle recommendations, supporting the need for culturally appropriate hypertension care [101].

## Conclusions

A growing body of evidence shows that many African migrant groups experience a higher burden of CVD and its risk factors. However, there is substantial heterogeneity based on country of origin, host country, reason for migration, duration of stay, sex, and age among others. This variation is likely driven by differences in pre- and post-migration factors, including early life factors, SES, work and occupation, neighbourhood environment, social support, cultural factors, access to health care, genetics, and epigenetics. In addition, the majority of studies originate from a handful of European and North American countries. Future studies should aim to fill the existing gaps by including diverse African migrant populations in diverse settings, with a focus on longitudinal studies that can assess temporal changes. To advance our understanding of the drivers and mechanisms, these studies will need to collect detailed data on lifestyle, sociocultural, environmental, as well as genetic factors. Knowledge generated from these types of studies is essential to develop targeted diagnostic, preventive, and treatment strategies. Addressing the CVD burden in African migrants requires a comprehensive approach that considers the multifaceted nature of the effects of migration on health. Through targeted research and intervention strategies, it is possible to mitigate CVD risk and improve health outcomes for African migrants globally.

## Data Availability

No datasets were generated or analysed during the current study.

## Key References

• Razieh C, Zaccardi F, Miksza J, Davies MJ, Hansell AL, Khunti K, et al. Differences in the risk of cardiovascular disease across ethnic groups: UK Biobank observational study. Nutr Metab Cardiovasc Dis. 2022;32(11):2594 − 602. **Large study exploring differences in CVD risk and its risk factors for African and other ethnic minority populations living in the UK.**

• Looti AL, Ovbiagele B, Markovic D, Towfighi A. All-Cause, Cardiovascular, and Stroke Mortality Among Foreign-Born Versus US-Born Individuals of African Ancestry. J Am Heart Assoc. 2023;12(9):e026331. **This study showed that the CVD risk profile of recent African migrants differs from other African-ancestry groups in the US**,** showing that findings from African Americans**,** for example**,** should not be extrapolated to African migrants.**

•• Mensah D, Ogungbe O, Turkson-Ocran R-AN, Onuoha C, Byiringiro S, Nmezi NA, et al. The Cardiometabolic Health of African Immigrants in High-Income Countries: A Systematic Review. International Journal of Environmental Research and Public Health. 2022;19(13):7959. **A comprehensive systematic review providing pooled estimates for the burden of cardiometabolic risk factors among African migrants in high-income countries.**

• Ismail SU, Asamane EA, Osei-Kwasi HA, Boateng D. Socioeconomic Determinants of Cardiovascular Diseases, Obesity, and Diabetes among Migrants in the United Kingdom: A Systematic Review. International journal of environmental research and public health. 2022;19(5):3070. **This systematic review examines the relationship between socioeconomic determinants and CVD risk in migrants**,** highlighting their varying impact across migrant subgroups.**

•• Chilunga FP, Henneman P, Venema A, Meeks KAC, Gonzalez JR, Ruiz-Arenas C, et al. DNA Methylation as the Link between Migration and the Major Noncommunicable Diseases: the RODAM Study. Epigenomics. 2021;13(9):653 − 66. **This is the first and only study examining the role of epigenetics in migration and health.**
